# Supporting Cells and Their Potential Roles in Cisplatin-Induced Ototoxicity

**DOI:** 10.3389/fnins.2022.867034

**Published:** 2022-04-27

**Authors:** Sofia Waissbluth, Juan Cristóbal Maass, Helmuth A. Sanchez, Agustín D. Martínez

**Affiliations:** ^1^Department of Otolaryngology, Pontificia Universidad Católica de Chile, Santiago, Chile; ^2^Department of Otolaryngology, Hospital Clínico de la Universidad de Chile, Santiago, Chile; ^3^Centro Interdisciplinario de Neurociencia de Valparaíso, Facultad de Ciencias, Instituto de Neurociencia, Universidad de Valparaíso, Valparaíso, Chile

**Keywords:** cisplatin, ototoxicity, supporting cells, gap junction, connexin, hemichannels

## Abstract

Cisplatin is a known ototoxic chemotherapy drug, causing irreversible hearing loss. Evidence has shown that cisplatin causes inner ear damage as a result of adduct formation, a proinflammatory environment and the generation of reactive oxygen species within the inner ear. The main cochlear targets for cisplatin are commonly known to be the outer hair cells, the stria vascularis and the spiral ganglion neurons. Further evidence has shown that certain transporters can mediate cisplatin influx into the inner ear cells including organic cation transporter 2 (OCT2) and the copper transporter Ctr1. However, the expression profiles for these transporters within inner ear cells are not consistent in the literature, and expression of OCT2 and Ctr1 has also been observed in supporting cells. Organ of Corti supporting cells are essential for hair cell activity and survival. Special interest has been devoted to gap junction expression by these cells as certain mutations have been linked to hearing loss. Interestingly, cisplatin appears to affect connexin expression in the inner ear. While investigations regarding cisplatin-induced hearing loss have been focused mainly on the known targets previously mentioned, the role of supporting cells for cisplatin-induced ototoxicity has been overlooked. In this mini review, we discuss the implications of supporting cells expressing OCT2 and Ctr1 as well as the potential role of gap junctions in cisplatin-induced cytotoxicity.

## Introduction

The cochlear sensory epithelium, also known as the organ of Corti, contains both hair cells (HCs) and supporting cells. The HCs, or sensory cells, are specialized and translate the mechanical energy of sound into neurophysiological signals. The supporting cells, on the other hand, are involved in the maintenance of the epithelium during hearing and development. Although they are not the actual transducers, they are essential, and without them, hearing would not be possible (Wan et al., 2013). In fact, some mutations linked to genetic deafness affect genes expressed in supporting cells but not in HCs, such as connexin (Cx) genes for gap junction formation (Forge et al., 1999; Martínez et al., 2009). There are approximately 15 supporting cells per each inner hair cell (IHC) and there are different types of supporting cells including: border, inner phalangeal, pillar, Deiters’ (outer phalangeal cells), and Hensen’s cells (Merchant et al., 2010). They span the whole thickness of the epithelium providing a rigid but vibratile backbone for the organ of Corti. In their luminal domain, they form tight unions with HCs, electrochemically isolating the endolymph, and enabling the endocochlear potential. However, at the basolateral domain, they are widely connected to HCs with important communicative, nutritive, and homeostatic functions (Wangemann, 2006; Zdebik et al., 2009). They can mediate recycling of neurotransmitters and ions, and also perform immunologic and housekeeping functions (Abrashkin et al., 2006; Bird et al., 2010). They can phagocyte or eject fragments of cells, debris, and even whole HCs from the epithelium that have been terminally injured, and produce a phalangeal scar to keep the integrity and electrochemical properties of the epithelium (Kelley et al., 1995; Żak et al., 2016). During organ development, supporting cells participate in cell patterning, polarity and synaptogenesis. There is evidence from other species that supporting cells can perform regenerative functions (Cotanche, 1987; Corwin and Cotanche, 1988). In mammals, although there is no spontaneous cochlear regeneration, it is possible to induce trans-differentiation or proliferation in supporting cells *in vitro* and *in vivo* (Chen and Segil, 1999; Löwenheim et al., 1999; Izumikawa et al., 2005; Sage et al., 2005; White et al., 2006; Yu et al., 2010; Yang et al., 2012; Mizutari et al., 2013; Atkinson et al., 2014; Cox et al., 2014; Silva and Maass, 2019). Additionally, supporting cells are more resistant than HCs and usually remain in the epithelium upon damage (McFadden et al., 2002, 2004; Raphael et al., 2007; deTorres et al., 2019). Thus, they are potential targets for regenerative therapies. Despite their importance, they have drawn much less attention and have not been studied as much as HCs (Wan et al., 2013; Waldhaus et al., 2015; Maass et al., 2016). Moreover, it is known that persistent or very severe damage to the sensory epithelium may destroy supporting cells and result in a flat epithelium, in which the normal columnar specialized epithelium is replaced by a non-specialized monolayer epithelium (McFadden et al., 2002, 2004; Raphael et al., 2007; Izumikawa et al., 2008). Several drugs can induce damage to the organ of Corti, however, their effects have been mostly characterized for HCs, and not for supporting cells (Anniko and Sobin, 1986; Slattery and Warchol, 2010).

In this article, we review the potential roles supporting cells may play in cisplatin-induced ototoxicity.

## Cisplatin-Induced Cytotoxicity

Cisplatin is a commonly used chemotherapeutic agent worldwide. It is a platinating agent that is used to treat various types of cancers (Gold and Raja, 2021). While it is quite efficient as a chemotherapeutic agent, it is known to cause various dose-limiting side effects which include nephrotoxicity and ototoxicity (Wensing and Ciarimboli, 2013). Cisplatin cytotoxicity derives mainly from its capacity to form irreversible DNA adducts, disrupting replication and transcription, and leading to cell death as the cell fails to repair itself (Jamieson and Lippard, 1999).

Cisplatin is a small, and highly reactive molecule and it is believed to enter the inner ear cells through passive diffusion and various transporters, mainly organic cation transporter 2 (OCT2)/solute carrier (SLC) 22A2, and copper transporter Ctr1 (Ciarimboli et al., 2010; More et al., 2010). Once inside the cell, it undergoes an aquation reaction and can bind irreversibly to DNA, RNA and proteins, which leads to cell death, mainly by apoptosis (Johnstone et al., 2016). Ototoxicity is believed to occur as a result of these adducts, a proinflammatory environment and the generation of reactive oxygen species (ROS) within the inner ear. Cisplatin increases the release of proinflammatory cytokines TNF-α, IL-1β and IL-6, and activates MAPKs and factor NF-κB which in turn, promote the expression of pro-inflammatory genes (So et al., 2008). It also activates signal transducer and activator of transcription family proteins, STAT1 and STAT6, which also promote an inflammatory response (Gentilin et al., 2019).

Reactive oxygen species play a major role in cisplatin-induced ototoxicity. They can activate NOX3, a NADPH oxidase highly expressed in the cochlea (Bánfi et al., 2004), which leads to lipid peroxidation and the accumulation of ROS. Cisplatin also depletes glutathione and antioxidant enzymes, which further increases lipid peroxidation (Rybak et al., 2007). As a result of the overwhelming oxidative stress, the cells undergo apoptosis (Sheth et al., 2017; Gentilin et al., 2019). Interestingly, a recent study has shown that following cisplatin exposure in a murine model, Nox3 expression is increased in supporting cells and outer hair cells (OHCs), especially at the basal turn of the cochlea, yet, OHCs but not supporting cells exhibited ROS-induced apoptosis from endogenously produced ROS and/or that of surrounding supporting cells (Mohri et al., 2021) indicating that somehow supporting cells are resistant to ROS-induced cell damage.

On the other hand, morphological analysis following cisplatin application shows that supporting cells exhibit signs of structural damage even before HC loss (Ramírez-Camacho et al., 2004). Moreover, phagocytosis of dead HCs by supporting cells seems to be impaired after cisplatin treatment (Monzack et al., 2015), indicating that supporting cells can be a direct target of cisplatin damage. The association between supporting cells and HC survival can be further supported by the finding that constitutive activation of PI3K-dependent survival signals in some supporting cells, by means of specific genetic ablation of Phosphatase and Tensin Homolog (PTEN), protects HCs from cisplatin damage (Jadali et al., 2017). This protection only occurs in the nearest neighboring HC to the supporting cell presenting greater activity for PI3K, probably through the activation of Checkpoint Kinase 1 (CHK1), which allows supporting cells to repair cisplatin-induced DNA damage (Jadali et al., 2017). Hence, the protection of supporting cells could indirectly protect HCs from cisplatin damage through the secretion of unknown extracellular signaling molecules or other cell-cell signaling molecules that activate HC survival programs.

## Cisplatin-Induced Hearing Loss

Clinically, cisplatin-induced ototoxicity presents as a high frequency sensorineural hearing loss that progresses toward the low frequencies (Sheth et al., 2017). Hearing loss is progressive and can also appear months to years after the end of the chemotherapy treatment (Einarsson et al., 2010; Waissbluth et al., 2018). Platinum has been shown to be retained in the inner ear after cisplatin chemotherapy and this has been suggested as one of the explanations for progressive hearing loss (Breglio et al., 2017). Patients often have tinnitus (Sakamoto et al., 2000; Dille et al., 2010; Frisina et al., 2016; Brooks and Knight, 2018) and in some cases, bilateral vestibulopathy (Prayuenyong et al., 2018).

An important subset of patients are pediatric patients because hearing loss can have debilitating long term effects including difficulties in learning, speech delays, and psychosocial impairment (Sheth et al., 2017). Another subset of high-risk patients are patients with cancers requiring concomitant head and neck radiotherapy, as this is an independent risk factor for developing hearing loss. Other risk factors include age (<5 years, >65 years), type of administration, renal function, cumulative cisplatin dose, concomitant use of other ototoxic medications and genetic predisposition (Paken et al., 2019). To date, there are no protective strategies or treatments for cisplatin-induced hearing loss that are FDA-approved. Despite its clinical relevance, the mechanisms by which cisplatin induces hearing loss are still under scrutiny, without clear evidence supporting a principal venue.

## Main Transporters Involved in Cisplatin Transport Into the Inner Ear

**FIGURE 1:**
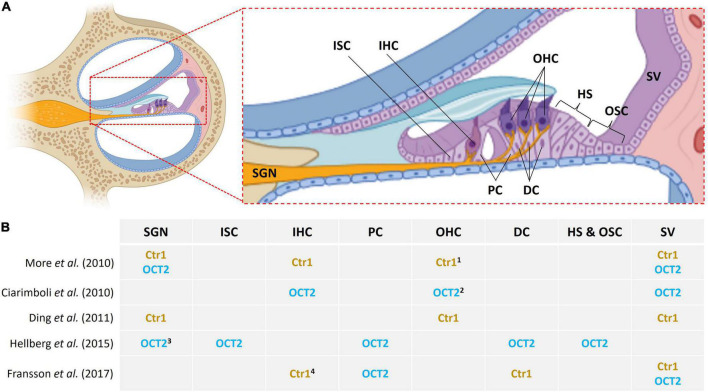
Location of Ctr1 and OCT2 in inner ear cells. **(A)** Midmodiolar cross section of the cochlea with emphasis on the organ of Corti. Different cell types are indicated as follows: SGN, spiral ganglion neurons; ISC, inner sulcus cells; IHC, inner hair cells; PC, pillar cells; OHC, outer hair cells; DC, Deiters’ cells; HS, Hensen’s cells; OSC, outer sulcus cells; SV, stria vascularis. **(B)** Variable and inconsistent localization of Ctr1 and OCT2 in the inner ear in various studies; ^1^ base of OHC, ^2^ apical pole of OHC, ^3^ SGN type 1, ^4^ IHC synapse area.

van Ruijven et al. (2004) described that cisplatin causes HC loss which is accompanied by protrusion of supporting cells into Nuel’s space and the tunnel of Corti, resulting in a disturbed microarchitecture of the organ of Corti. Thereafter, the same group proposed that the main inner ear targets for cisplatin toxicity are the OHCs, stria vascularis and the spiral ganglion neurons (SGN) (van Ruijven et al., 2005). The latter indicates that cisplatin may induce generalized damage to cochlear tissue more than a cell-type specific effect. Evidence shows that transporters that may mediate cisplatin influx include OCT2 and Ctr1, and they are expressed in cochlear tissue (Figure 1). However, the expression profiles for these transporters are not consistent in the literature. More et al. (2010) observed the presence of Ctr1 in the IHCs, OHCs, stria vascularis and SGN; and OCT2 in the stria vascularis and SGNs but absent from HCs, in 3–4 weeks mouse cochleae. Ding et al. (2011) found Ctr1 in HCs, epithelium of the stria vascularis, and SGN in postnatal day 3 rat pups while Fransson et al. (2017) observed expression in the IHC synapse area, Deiters’ cells and stria vascularis in guinea pigs.

On the other hand, Ciarimboli et al. (2010) observed OCT2 distributed throughout IHCs, at the apical pole of OHCs, and the stria vascularis but not in the SGN; they do, however, mention some expression in the inner spiral bundle fibers. They also looked into Ctr1; it was present in mouse cochleae but in lower expression as compared to OCT2, hence they decided not to perform further testing for Ctr1; they focused on OCT2 seen as it had greater expression by RT-PCR. Later on, Hellberg et al. (2015) found OCT2 in supporting cells and SGN but not HCs or the stria. They report OCT2 in Deiters’ cells, Hensen’s cells, outer and inner sulcus cells, and outer and inner pillar cells (Hellberg et al., 2015). Consistently, Fransson et al. (2017) also showed strong immunoreactivity for OCT2 in inner and outer pillar cells, and also found Ctr1 in Deiters’ cells and in the stria but not in OHCs.

Another interesting finding is the presence of ATP7A in the pillar cells (Ding et al., 2011). P-type ATPases, copper-transporting ATP7A and ATP7B, are believed to be involved in cisplatin sequestration and efflux (Safaei, 2006), and overexpression has been linked to cisplatin resistance in cancer cells (Samimi et al., 2004). Remarkably, although pillar cells express OCT2, they also express ATP7A and only limited uptake of cisplatin has been reported in supporting cells; while ATP7B was localized in the OHCs of postnatal day 3 rat pups (Ding et al., 2011).

## What About Gap Junctions?

Connexins (Cxs) are transmembrane proteins pivotal for cell communication as they assemble in hemichannels and gap junction channels (GJCs). Hemichannels connect the cell with its external *milieu* either in a paracrine or autocrine manner (Sáez and Leybaert, 2014). GJCs are aqueous pores through appositional plasma membranes in neighboring cells that connect their cytoplasm. Both hemichannels and GJCs allow the passage of molecules and ions that are essential to maintain cell homeostasis (Sáez et al., 2003) such as ATP, miRNA, glucose, NAD, second messengers and other signaling molecules (Kang et al., 2008; Zong et al., 2016). In physiological conditions, extracellular Ca^2+^ and the resting membrane potential keep hemichannels closed, and depending on the Cx isoform, hemichannels can be opened by diverse signaling mechanisms including phosphorylation, calcium-calmodulin, nitrosylation, etc. (Sáez and Leybaert, 2014).

Several Cx genes have been identified in the cochlea, including Cx26, Cx30, Cx31, Cx29, Cx43, and Cx45 (Forge et al., 2003a; Martínez et al., 2009). Although HCs do not express Cxs, supporting cells are highly interconnected through GJCs formed mainly by Cx26 and Cx30, forming functional heteromeric hemichannels and GJCs (Ahmad et al., 2003; Forge et al., 2003b; Sun et al., 2005; Verselis, 2019), with some reports indicating expression of Cx43 in rodent prenatal stages (Cohen-Salmon et al., 2004; Jagger and Forge, 2015). Mutations in Cx26 gene (GJB2) are responsible for 50% of genetic deafness causing sensorineural hearing loss due to cochlear malfunction (Kelsell et al., 1997; Martínez et al., 2009; García et al., 2015, 2016; Verselis, 2019). Cxs are involved in the regulation and recycling of K^+^ and pH maintenance, along with the passage of molecules such as ATP, IP3 and others between supporting cells (Zhao et al., 2005; Jagger and Forge, 2015; Verselis, 2019), maintaining the sensitivity and viability of HCs (Ramírez-Camacho et al., 2006; Zhu et al., 2013) and general homeostasis of the auditory sensory epithelium.

In certain types of cancer cells, communication through GJCs allows the spread of toxic signals to adjoining cells in response to cisplatin treatment, potentiating cell death, a process named “Bystander effect” (Jensen and Glazer, 2004; Arora et al., 2018). A similar mechanism has been proposed for cisplatin-induced toxicity in the organ of Corti. A report showed that inhibition of GJCs with 18α-GA, a non-selective blocker, reduced cisplatin-induced apoptosis of auditory HCs (Kim et al., 2014). They suggest Cx43 may play a proapoptotic role (Kim et al., 2016) as treatment of HEI-OC1 cells, an undifferentiated organ of Corti progenitor cell line, with Cx43 siRNA, present greater cell viability compared to control cells during *in vitro* cisplatin treatment (Kim et al., 2014). However, a recent study contradicts this “Bystander effect” and the necessity of Cx43 function in cisplatin-induced propagation of death signals in the organ of Corti (Abitbol et al., 2020). In this report, researchers found that in organotypic cochlear cultures from two Cx43-mutant mouse strains expressing Cx43 mutations characterized by moderate (Cx43I130T/+) or severe (Cx43G60S/+) reduction of Cx43 GJC function, cisplatin-induced HC apoptosis was similar to wild type cochlear cultures. In addition, the inhibition of GJC with carbenoxolone did not modify cisplatin-induced HC death (Abitbol et al., 2020). However, as mentioned early, in the mature inner ear, Cx43 is not expressed in supporting cells (Jagger and Forge, 2015).

On the other hand, in some conditions, GJCs can dilute toxic signals induced by cisplatin, which can lead to protection (Hong et al., 2012). In fact, it has been proposed that gap junctions protect non-cancer cells from cisplatin toxicity while enhancing it in tumor cells (Hong et al., 2012; Zhang et al., 2015). Protective signals such as cAMP can pass through gap junctions spreading the transcellular activation of cAMP/PKA/CREB signaling, thereby reducing cisplatin toxicity in organ of Corti cells (Kim et al., 2021). Supporting previous findings, gap junction enhancers, all-*trans* retinoic acid and quinoline, potentiate the effects of forskolin induced-cAMP production on cell survival via activation of cAMP/PKA/CREB (Kim et al., 2021). Moreover, mice treated with cisplatin exhibited damage to the stria vascularis and reduced endocochlear potentials. This was associated with decreasing expressions of Cx26 and Cx43 in marginal and basal cells of the stria vascularis (Zhang et al., 2020). The latter result is consistent with findings showing that the deletion or mutation of the Cx26 gene negatively impacts the endocochlear potential (Mei et al., 2017). In addition, Wang et al. (2010) found that high concentrations of cisplatin or oxaliplatin inhibit the activity of GJCs formed by Cx26 and Cx32 in HeLa cells. Furthermore, using reconstituted connexin-containing liposomes (immunopurified Cx26/Cx32 hemichannels), the authors found that cisplatin reduces the activity of the purified hemichannels, suggesting that cisplatin may interfere directly with channels made by Cx26 and Cx32. Interestingly, the concentration range for cisplatin used in this study (0.5–7.5 mg/ml) is similar to the cisplatin plasma concentration found in patients during cancer treatment. Moreover, prolonged treatment with cisplatin (48 h) reduces the expression of Cx26 in transfected HeLa cells. Therefore, the inhibition of GJCs by cisplatin and oxaliplatin decreases the cytotoxicity of these compounds, thereby generating a form of resistance to these antitumor agents (Wang et al., 2010). However, in the cochlea, the possible inhibition of gap junctions induced by cisplatin may contribute to HC death as gap junctions are critical for homeostasis and cochlear function.

## Discussion

The precise pathophysiology of cisplatin-induced ototoxicity remains unknown. What we do know is that oxidative stress, inflammation and DNA adducts lead to cell death in the cochlea. It appears OCT2 and Ctr1, which would allow cisplatin influx, are expressed in supporting cells, yet the OHCs die initially; and that the distribution of potential transporters is variable depending on experiment/cells/species studied. Most interestingly, if OHCs are highly susceptible to cisplatin, a logical question would be: are these cells accumulating more cisplatin than other structures in the cochlea? Well, it appears they don’t. Immunohistochemical detection of cisplatin-DNA adducts has been observed in the nuclei of most cells in the organ of Corti and the lateral wall after cisplatin administration (van Ruijven et al., 2005). Using laser ablation coupled to ICP-MS, concentrations of platinum within the organ of Corti were indistinguishable from neighboring tissue, suggesting no specific accumulation of cisplatin within HCs (Breglio et al., 2017). Cisplatin does, however, accumulate consistently in the stria vascularis (van Ruijven et al., 2005; Thomas et al., 2006; Breglio et al., 2017), and it has been proposed that disruption of cochlear fluid homeostasis could lead to HC apoptosis (Prayuenyong et al., 2021).

**FIGURE 2:**
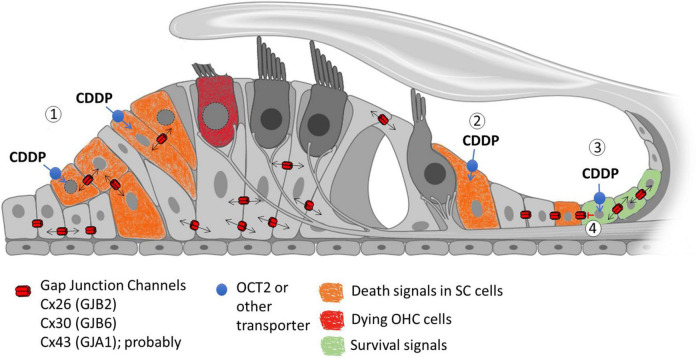
Possible role of Gap Junction Channels (GJC) in the propagation of cisplatin-induced cellular signals. (1) Cisplatin (CDDP) can potentially enter supporting cells (SCs) through non-selective transporters such as OCT2. Inside the cells, cisplatin induces toxicity by damaging DNA and mitochondria, producing ROS and other death signals that can propagate through GJC, spreading cell death signals through the sensory epithelium (Bystander effect). (2) Cisplatin can potentially enter SCs that lack intercellular communication through gap junctions, which restrict toxicity only to these cells. (3) Alternatively, GJC can potentially allow the transmission of protective signals that can reduce cell death between the coupled cells affected by cisplatin. (4) The loss of gap junction communication induced by cisplatin could also induce loss of cell viability in SCs.

Another possibility is that HCs are somehow exposed to cell death signals that may be from surrounding cells. We know Cxs are present in all types of non-sensory cells in the cochlea and that they are essential for hearing (Wu et al., 2019). There is also evidence that they can spread either toxic or protective signals generated by cisplatin to adjoining cells (Figure 2). However, contradictory evidence on the exact role of gap junctional intercellular communication in cisplatin-induced ototoxicity impedes a mechanistic model. There is variable expression of Cxs in the organ of Corti and it has been demonstrated that cisplatin can decrease the activity and expression of Cxs. Hence, a decreased expression in the supporting cells, or lateral wall, could perhaps be involved in OHC death.

While the commonly known areas of the cochlea that are affected by cisplatin are the OHCs, stria vascularis and SGN, greater research efforts should be focused on the supporting cells as they are essential for HC activity and survival. Further research is needed to better understand the pathophysiology of cisplatin-induced ototoxicity, cisplatin inner ear trafficking, as well as the functions of gap junctions in the cochlea.

## Author Contributions

All authors listed have made a substantial, direct, and intellectual contribution to the work, and approved it for publication.

## Conflict of Interest

The authors declare that the research was conducted in the absence of any commercial or financial relationships that could be construed as a potential conflict of interest.

## Publisher’s Note

All claims expressed in this article are solely those of the authors and do not necessarily represent those of their affiliated organizations, or those of the publisher, the editors and the reviewers. Any product that may be evaluated in this article, or claim that may be made by its manufacturer, is not guaranteed or endorsed by the publisher.
